# Protein Kinase C Inhibitors Reduce SARS-CoV-2 Replication in Cultured Cells

**DOI:** 10.1128/spectrum.01056-22

**Published:** 2022-08-24

**Authors:** Changbai Huang, Fei Feng, Yongxia Shi, Wenhui Li, Ziqiao Wang, Yunkai Zhu, Shuai Yuan, Dandan Hu, Jun Dai, Qiqi Jiang, Rong Zhang, Chao Liu, Ping Zhang

**Affiliations:** a Key Laboratory of Tropical Diseases Control (Sun Yat-sen University), Ministry of Education, Guangzhou, China; b Department of Immunology, Zhongshan School of Medicine, Sun Yat-sen University, Guangzhou, China; c Key Laboratory of Medical Molecular Virology (MOE/NHC/CAMS), School of Basic Medical Sciences, Shanghai Medical College, Fudan University, Shanghai, China; d Health and Quarantine Institute, Guangzhou Customs Technology Centre, Guangzhou, China; e Department of Microbiology, Zhongshan School of Medicine, Sun Yat-sen University, Guangzhou, China; University of Arizona

**Keywords:** SARS-CoV-2, PKC, replicon, translation

## Abstract

Infection by severe acute respiratory syndrome-related coronavirus 2 (SARS-CoV-2) has posed a severe threat to global public health. The current study revealed that several inhibitors of protein kinases C (PKCs) possess protective activity against SARS-CoV-2 infection. Four pan-PKC inhibitors, Go 6983, bisindolylmaleimide I, enzastaurin, and sotrastaurin, reduced the replication of a SARS-CoV-2 replicon in both BHK-21 and Huh7 cells. A PKCδ-specific inhibitor, rottlerin, was also effective in reducing viral infection. The PKC inhibitors acted at an early step of SARS-CoV-2 infection. Finally, PKC inhibitors blocked the replication of wild-type SARS-CoV-2 in ACE2-expressing A549 cells. Our work highlights the importance of the PKC signaling pathway in infection by SARS-CoV-2 and provides evidence that PKC-specific inhibitors are potential therapeutic agents against SARS-CoV-2.

**IMPORTANCE** There is an urgent need for effective therapeutic drugs to control the pandemic caused by severe acute respiratory syndrome-related coronavirus 2 (SARS-CoV-2). We found that several inhibitors of protein kinases C (PKCs) dramatically decrease the replication of SARS-CoV-2 in cultured cells. These PKC inhibitors interfere with an early step of viral infection. Therefore, the rapid and prominent antiviral effect of PKC inhibitors underscores that they are promising antiviral agents and suggests that PKCs are important host factors involved in infection by SARS-CoV-2.

## INTRODUCTION

Severe acute respiratory syndrome-related coronavirus 2 (SARS-CoV-2) is the causative agent of coronavirus disease 2019 (COVID-19). Its infection has caused a global health emergency, with more than 546 million cases and 6.3 million deaths as of July 2022. Although some patients showed mild symptoms, a significant number of patients develop acute respiratory distress syndrome and fibrosis (1). Vaccination programs against SARS-CoV-2 are ongoing, whether these vaccines provide a long-term protective effect remains unknown. So far, the U.S. Food and Drug Administration (FDA) has approved a few anti-SARS-CoV-2 agents, including remdesivir, molnupiravir, and nirmatrelvir-ritonavir (Paxlovid), for the treatment of COVID-19 (2–4). Unfortunately, these drugs showed limited efficacy in clinical use (5). Therefore, there is still an urgent need for the development or repurposing of antiviral drugs.

The genome of SARS-CoV-2 is a positive single-stranded RNA, about 30 kb in length. The viral genome encodes replicases, 4 structural proteins (spike, envelope, membrane, and nucleocapsid proteins), 16 nonstructural proteins (NSPs), and 9 accessory proteins (6). As a member of the RNA viruses, the genome of SARS-CoV-2 is prone to mutation during replication (7), leading to the potential evasion of antiviral drugs targeting viral proteins. Therefore, screening of antiviral agents targeting host factors involved in viral replication becomes a topic of interest.

A family of closely related cellular kinases, protein kinases C (PKCs), has been implicated in the regulation of infection by a variety of viruses. The PKC family is classified into three subfamilies: conventional (α, β, and γ), novel (δ, ε, η, and θ) (8, 9), and atypical (μ, ξ, and ι) (10, 11). Upon binding to phosphatidylserine, PKCs are activated and then translocate from the cytoplasm to the plasma membrane. PKCs play important roles in multiple cellular processes such as apoptosis, differentiation, proliferation, and adhesion (12). PKCs participate in the replication of influenza virus (13), hepatitis E virus (14), human immunodeficiency virus type 1 (HIV-1) (15), minute virus of mice (MVM) (16), West Nile virus (17), chikungunya virus (CHIKV) (18), and dengue virus (DENV) (19). SARS-CoV activates the PKC members (20), implying that they might be involved in SARS-CoV-2 infection. An *in vitro* study revealed that bisindolylmaleimide IX, a PKC inhibitor, targets the viral protease 3CL^pro^ (21). Nonetheless, the impact of PKCs on SARS-CoV-2 infection has not been experimentally tested in an infection model.

In current study, we employed four pan-PKC inhibitors, Go 6983, bisindolylmaleimide I, enzastaurin, and sotrastaurin, and examined their impact on viral replication using a SARS-CoV-2 replicon system. Our data showed that these PKC inhibitors reduced the viral replication. A specific inhibitor of PKCδ, rottlerin, also impaired viral replication. Three PKC inhibitors confer antiviral activity against wild-type SARS-CoV-2. These findings support that PKC inhibitors could be promising therapeutic drugs.

## RESULTS

### PKC inhibitors reduce the replication of SARS-CoV-2 in BHK-21 cells.

**FIG 1:**
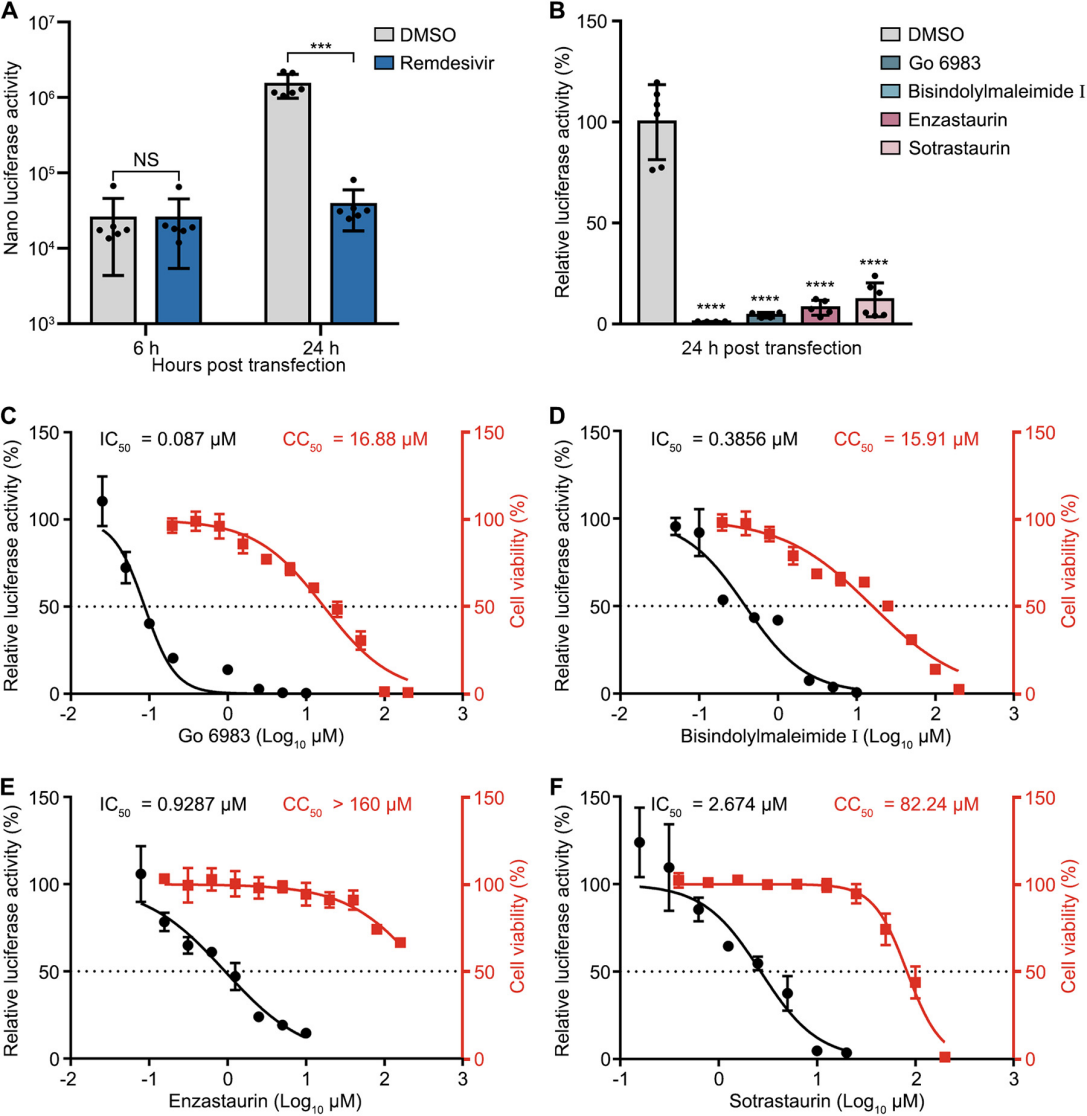
PKC inhibitors reduce the replication of SARS-CoV-2 in BHK-21 cells. (A and B) Luciferase activity of the replicon system. BHK-21 cells were pretreated with DMSO, remdesivir (A), or PKC inhibitors (Go 6983, bisindolylmaleimide I, enzastaurin, or sotrastaurin) (B) for 1 h, followed by replicon RNA transfection. The luciferase activities were determined at 6 or 24 h posttransfection. (C to F) IC_50_ and CC_50_ values of Go 6983 (C), bisindolylmaleimide I (D), enzastaurin (E), and sotrastaurin (F) in BHK-21 cells. IC_50_ and CC_50_ values are indicated above the curves. The cytotoxic effects of each drug at the indicated concentrations were determined by a CCK8 cell viability assay at 24 h. CC_50_ values were calculated using Prism software. Data are presented as means ± standard deviations (SD) from three experiments; *P* values were calculated by unpaired, two-tailed Student’s *t* tests or ANOVA with Dunnett’s multiple-comparison test (***, *P* < 0.001; ****, *P* < 0.0001; NS, not significant).

To examine whether PKC inhibitors affect the replication of SARS-CoV-2, we utilized a SARS-CoV-2 replicon, which encodes viral NS proteins fused with a nanoluciferase reporter, replacing the structural proteins and several accessory proteins. We first tested the susceptibility of the SARS-CoV-2 replicon to remdesivir, a well-known anti-SARS-CoV-2 drug that inhibits viral RNA synthesis (5). BHK-21 cells were pretreated with dimethyl sulfoxide (DMSO) or remdesivir for 1 h, followed by replicon RNA transfection. The luciferase activity was measured at 6 h and 24 h posttransfection. As expected, remdesivir treatment did not alter the luciferase activity at 6 h, which indicates protein translation, but significantly reduced the luciferase activity at 24 h, which represents RNA synthesis (Fig. 1A), validating that our replicon system can be employed for drug screening.

Next, we selected four pan-PKC inhibitors, Go 6983, bisindolylmaleimide I, enzastaurin, and sotrastaurin (chemical structures are shown in Fig. S1 in the supplemental material), all of which inhibit several PKC isozymes, including PKCα, PKCβ, and PKCγ (22–25). BHK-21 cells were pretreated with the inhibitor for 1 h, followed by the transfection of replicon RNA. As shown in Fig. 1B, all PKC inhibitor treatments dramatically reduced the replication levels of SARS-CoV-2. The relative luciferase activities in cells treated with Go 6983, bisindolylmaleimide I, enzastaurin, and sotrastaurin were about 99%, 96%, 92%, and 89% lower than that in DMSO-treated cells, respectively. The 50% inhibitory concentrations (IC_50_s) of Go 6983, bisindolylmaleimide I, enzastaurin, and sotrastaurin were 0.087 μM, 0.3856 μM, 0.9287 μM, and 2.674 μM, respectively (Fig. 1C to F), indicative of the potent antiviral activity of these PKC inhibitors in BHK-21 cells. The 50% cytotoxicity concentrations (CC_50_s) of Go 6983, bisindolylmaleimide I, enzastaurin, and sotrastaurin were 16.88 μM, 15.91 μM, 160 μM, and 82.24 μM, respectively (Fig. 1C to F). Although Go 6983 and bisindolylmaleimide I displayed some cytotoxic effects at high concentrations, their CC_50_ values (16.88 μM and 15.91 μM, respectively) were much higher than their IC_50_ values (0.087 μM and 0.3856 μM, respectively). Overall, these data indicated that these pan-PKC inhibitors are potent antiviral compounds against SARS-CoV-2.

### PKC inhibitors confer anti-SARS-CoV-2 activity in human Huh7 cells.

**FIG 2:**
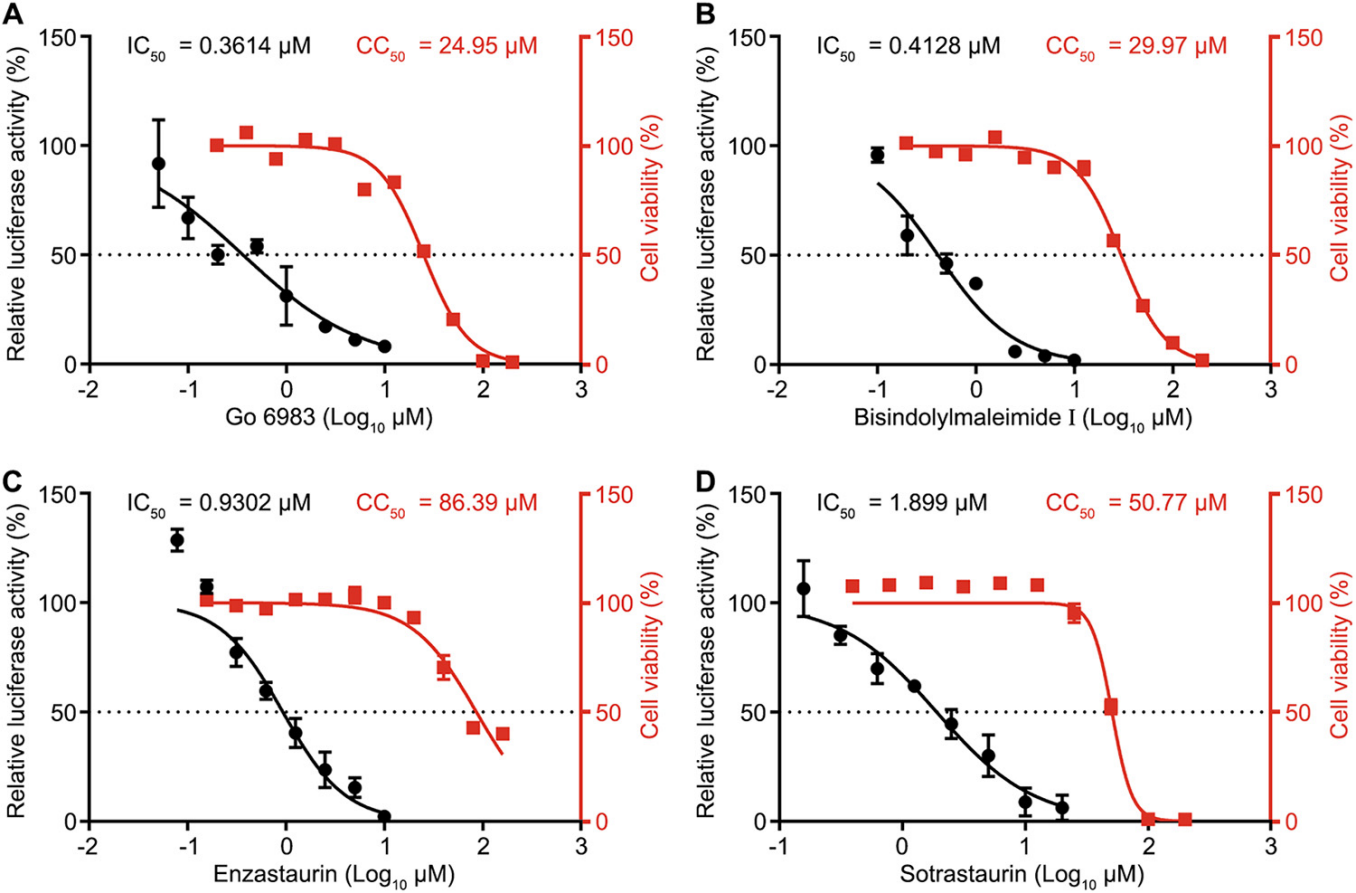
PKC inhibitors confer anti-SARS-CoV-2 activity in Huh7 cells. Shown are IC_50_ and CC_50_ values of Go 6983 (A), bisindolylmaleimide I (B), enzastaurin (C), and sotrastaurin (D) in human Huh7 cells. IC_50_ and CC_50_ values are indicated above the curves. The luciferase activities of the SARS-CoV-2 replicon were determined at 24 h posttransfection. The cytotoxic effects of each drug at the indicated concentrations were determined by a CCK8 cell viability assay at 24 h. CC_50_ values were calculated using Prism software. Data are presented as means ± SD from three experiments.

Next, we tested whether PKC inhibitors affect viral replication in human Huh7 cells. The IC_50_ values of Go 6983, bisindolylmaleimide I, enzastaurin, and sotrastaurin were 0.3614 μM, 0.4128 μM, 0.9302 μM, and 1.899 μM, respectively (Fig. 2A to D), indicating that they are also effective in Huh7 cells. The CC_50_ values of Go 6983, bisindolylmaleimide I, enzastaurin, and sotrastaurin were 24.95 μM, 29.97 μM, 86.39 μM, and 50.77 μM, respectively (Fig. 2A to D). Taken together, these PKC inhibitors displayed antiviral activity and limited cytotoxicity in human Huh7 cells.

### The PKCδ inhibitor rottlerin impairs the replication of SARS-CoV-2.

**FIG 3:**
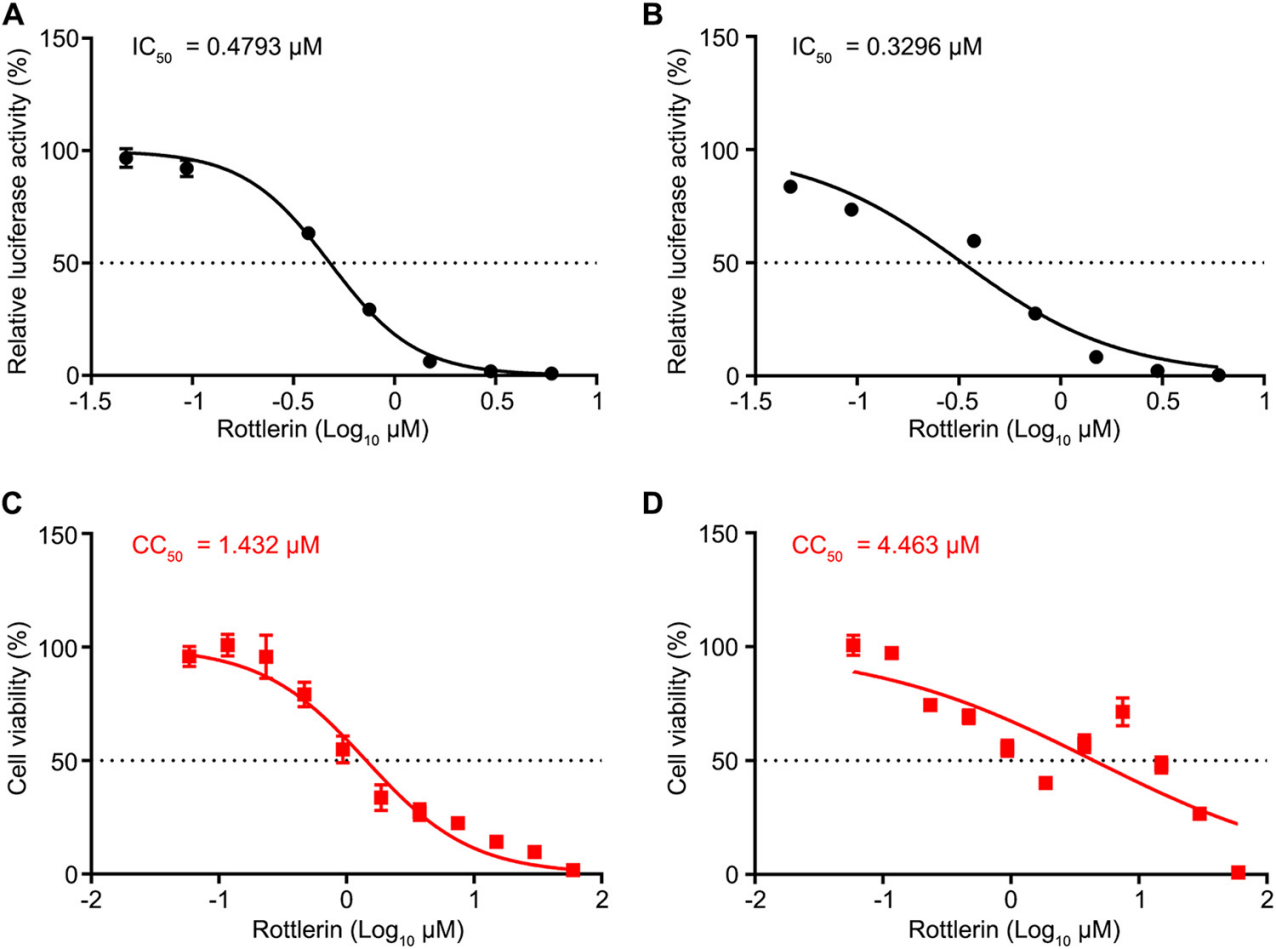
A PKCδ inhibitor impairs the replication of the SARS-CoV-2 replicon. (A and B) IC_50_s of rottlerin in BHK-21 (A) and human Huh7 (B) cells. IC_50_ values are indicated above the curves. (C and D) CC_50_s of rottlerin in BHK-21 (C) and human Huh7 (D) cells. The cytotoxic effects of each drug at the indicated concentrations were determined by a CCK8 cell viability assay at 24 h. CC_50_ values were calculated using Prism software and are indicated above the curves. Data are presented as means ± SD from three experiments.

The above-described data showed that SARS-CoV-2 was more susceptible to Go 6983 than to bisindolylmaleimide I, and the spectra of Go 6983 and bisindolylmaleimide I mostly overlapped, except that Go 6983 inhibits PKCδ, while bisindolylmaleimide I does not (22, 23). Therefore, we hypothesized that PKCδ might play a role in the replication of SARS-CoV-2. To test this hypothesis, we utilized rottlerin (the chemical structure is shown in Fig. S1), which is more selective for PKCδ than other PKC isozymes (26). Rottlerin treatment alone showed antiviral activity in both BHK-21 cells (IC_50_ = 0.4793 μM) and Huh7 cells (IC_50_ = 0.3296 μM) (Fig. 3A and B). The cytotoxicity of rottlerin was higher than those of the four above-described pan-PKC inhibitors (Fig. 3C and D). These data suggested that PKCδ is involved in the replication of SARS-CoV-2.

### PKC inhibitors function at an early step of SARS-CoV-2 replication.

**FIG 4:**
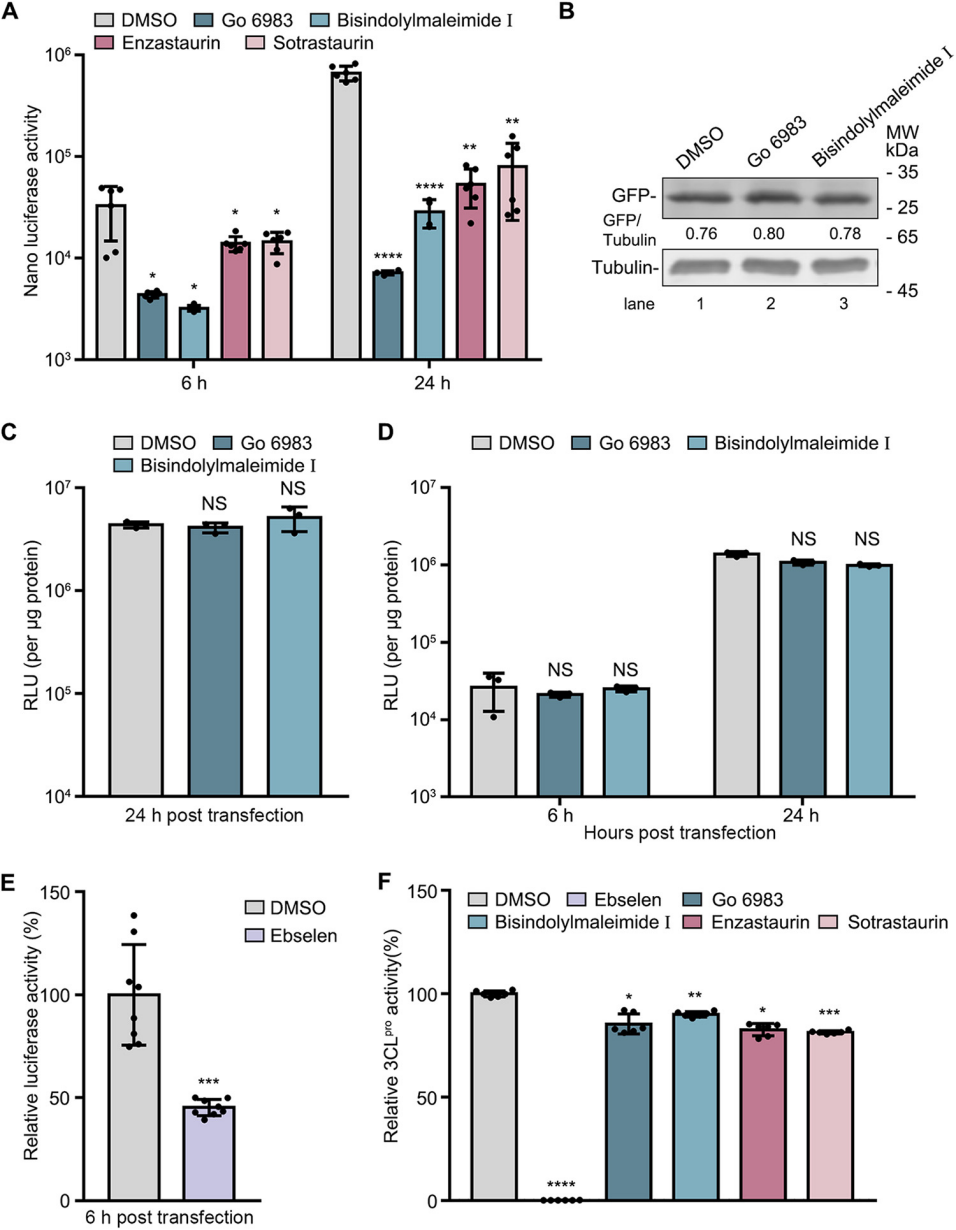
PKC inhibitors play an antiviral role during the early stage of SARS-CoV-2 replication. (A to E) Effect of PKC inhibitors on protein translation. BHK-21 cells were pretreated with DMSO or the indicated inhibitors for 1 h, followed by transfection with SARS-CoV-2 replicon RNA, GFP- or *Renilla* luciferase-expressing plasmids, or ZIKV replicon RNA. Luciferase assays or Western blot assays were performed at the indicated hours after transfection. MW, molecular weight; RLU, relative luciferase units. (F) Effect of PKC inhibitors on 3CL^pro^ activity. The protein activities were determined using a 2019 nCoV M^pro^/3CL^pro^ inhibitor screening kit. SARS-CoV-2 3CL^pro^, the substrate, and the inhibitors were incubated at 37°C for 5 min, and luciferase activities were measured at 490 nm using a multimode plate reader. Ebselen was used as a positive control. Data are presented as means ± SD from three experiments. *P* values were calculated by ANOVA with Dunnett’s multiple-comparison test or unpaired, two-tailed Student’s *t* tests (*, *P* < 0.05; **, *P* < 0.01; ***, *P* < 0.001; ****, *P* < 0.0001; NS, not significant).

To determine at which step the PKC inhibitors function, we monitored the kinetic luciferase activities of the replicon in BHK-21 cells. Cells were pretreated with PKC inhibitors and then transfected with replicon RNA. The cells were collected at 6 h and 24 h for the determination of luciferase activity. The relative luciferase activity at 6 h posttransfection is an indicator of protein translation or cleavage of the polyprotein, and at 24 h posttransfection, it indicates RNA replication. The luciferase activities in cells treated with Go 6983, bisindolylmaleimide I, enzastaurin, and sotrastaurin were constantly lower than those in DMSO-treated cells at all tested time points (Fig. 4A).

As PKC inhibitors reduced the luciferase activity of the replicon starting from 6 h, they probably block the protein translation of SARS-CoV-2 or the cleavage of the polyprotein. To test whether these inhibitors specifically block viral protein translation, we compared the expression levels of ectopically expressed green fluorescent protein (GFP) or *Renilla* luciferase in the absence and presence of the PKC inhibitor. At 24 h posttransfection, the GFP protein levels or relative *Renilla* luciferase activities were determined by a Western blot or luciferase assay. The data showed that the GFP levels and *Renilla* luciferase activities in the Go 6983- or bisindolylmaleimide I-treated cells were comparable to those in the DMSO-treated cells (Fig. 4B and C), suggesting that the protein translation of plasmids was not affected by PKC inhibitors. Next, to test whether the PKC inhibitors specifically block the protein translation of SARS-CoV-2, we utilized a Zika virus (ZIKV) replicon (27). The data showed that Go 6983 and bisindolylmaleimide I barely affected the replication levels of the ZIKV replicon at both 6 and 24 h posttransfection (Fig. 4D), indicating that PKC inhibitors do not broadly inhibit viral translation and replication.

To test whether the PKC inhibitors affect the cleavage of the viral polyprotein, we utilized ebselen, which targets SARS-CoV-2 3CL^pro^, as a positive control (28, 29). As expected, ebselen treatment reduced the luciferase activity of the SARS-CoV-2 replicon at 6 h (Fig. 4E). To explore whether the PKC inhibitors downregulate viral replication by targeting 3CL^pro^, we examined their impact on 3CL^pro^ activity using a 2019 nCoV (novel coronavirus) M^pro^/3CL^pro^ inhibitor screening kit. As shown in Fig. 4F, ebselen dramatically reduced the SARS-CoV-2 3CL^pro^ protease activity. In contrast, PKC inhibitors barely altered the 3CL^pro^ activity, indicating that they do not directly target 3CL^pro^. These data suggested that the PKC inhibitors function at an early step of SARS-CoV-2 replication. In addition, we tested whether they act at the entry step of SARS-CoV-2 using a pseudovirus model. Cells were pretreated with DMSO or the PKC inhibitors for 1 h and infected with pseudovirus. At 16 h postinfection (p.i.), the luciferase activity of pseudovirus, which indicates the level of entry of pseudovirus, was measured. The luciferase activities in Go 6983-, bisindolylmaleimide I-, enzastaurin-, and sotrastaurin-treated cells were about 35%, 24%, 42%, and 55% of those in the DMSO-treated cells, respectively (Fig. S2), suggesting that these PKC inhibitors might also act at the entry step of SARS-CoV-2.

### PKC inhibitors confer antiviral activity in a virus infection model.

**FIG 5:**
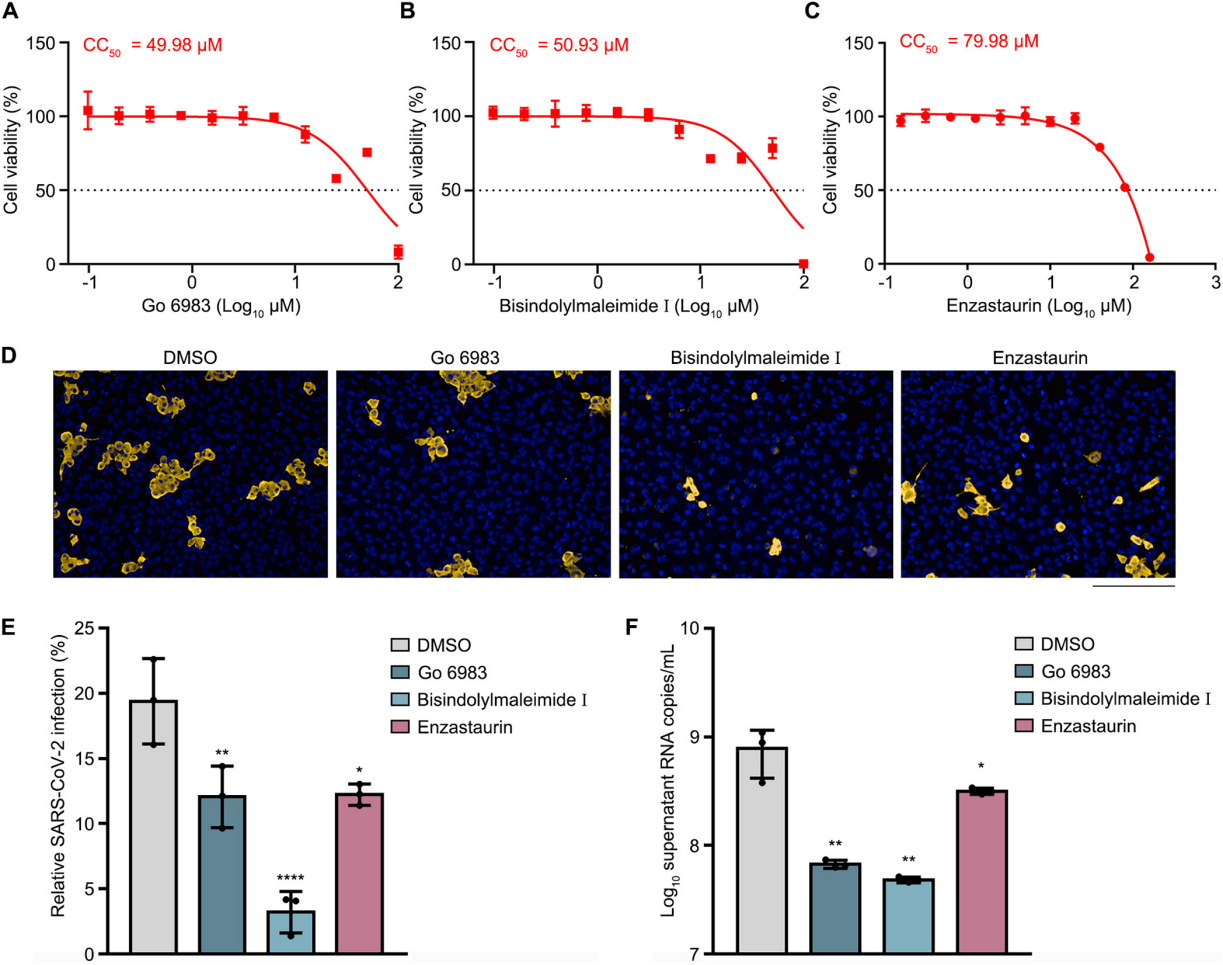
Replication of wild-type SARS-CoV-2 was impaired by PKC inhibitors. (A to C) CC_50_s of Go 6983 (A), bisindolylmaleimide I (B), and enzastaurin (C) in A549-ACE2 cells. The cytotoxic effects of each drug at the indicated concentrations were determined by a CCK8 cell viability assay. CC_50_ values are indicated above the curves. (D to F) Effect of PKC inhibitors on infection by SARS-CoV-2. A549-ACE2 cells were treated with DMSO, Go 6983, bisindolylmaleimide I, or enzastaurin for 1 h, followed by virus infection. At 24 h p.i., cells or supernatants were harvested for an immunofluorescence assay (D and E) or a qRT-PCR assay (F). Anti-SARS-CoV-2 N antibody was used to indicate the cells infected by virus (gold). The nucleus was stained with DAPI (blue). Representative images from three independent experiments are shown. Bar, 200 μm. The percentages of viral N-positive cells were calculated. Statistical analysis was carried out to reveal the proportion of cells infected by SARS-CoV-2 (E). qRT-PCR data are presented as means ± SD from three experiments. *P* values were calculated by ANOVA with Dunnett’s multiple-comparison test (*, *P* < 0.05; **, *P* < 0.01; ****, *P* < 0.0001).

Finally, we chose the three most potent PKC inhibitors, namely, Go 6983, bisindolylmaleimide I, and enzastaurin, to test their impact on the replication of wild-type SARS-CoV-2. The CC_50_s of Go 6983, bisindolylmaleimide I, and enzastaurin in ACE2-expressing A549 (A549-ACE2) cells were 49.98 μM, 50.93 μM, and 79.98 μM, respectively (Fig. 5A to C). Next, A549-ACE2 cells were pretreated with mock or the PKC inhibitor for 1 h, followed by SARS-CoV-2 infection at a multiplicity of infection (MOI) of 0.8. The cells and supernatants were harvested at 24 h p.i. for an immunofluorescence assay (IFA) using anti-N protein or quantitative real-time PCR (qRT-PCR) analysis. Treatment with the PKC inhibitors significantly reduced the percentages of N-positive cells (12% for Go 6983, 3% for bisindolylmaleimide I, and 12% for enzastaurin, versus 20% for DMSO) (Fig. 5D and E). Consistently, the viral RNA levels in the Go 6983-, bisindolylmaleimide I-, and enzastaurin-treated supernatants were about 12-, 16-, and 3-fold lower than those in the DMSO-treated cells (Fig. 5F). These data indicated that these compounds maintain a protective effect against infection with wild-type SARS-CoV-2, among which bisindolylmaleimide I confers the most potent activity.

## DISCUSSION

As SARS-CoV-2 is wreaking havoc around the world, the search for effective drugs is an urgent need for therapeutic application. In this report, we identified five PKC inhibitors, Go 6983, bisindolylmaleimide I, enzastaurin, sotrastaurin, and rottlerin, as potent antiviral compounds against SARS-CoV-2 infection.

To combat RNA viruses with high mutation rates, antiviral drugs targeting host factors might provide better protection than virus-targeted therapeutics, while the administration of host-targeted drugs may cause various side effects. Fortunately, PKC inhibitors have been developed for clinical use and have shown a good safety profile, as PKCs are closely associated with diverse diseases (30). For example, enzastaurin, a PKC inhibitor intended for the treatment of solid and hematological cancers, has gone through phase III clinical trials. Consistently, our work revealed that Go 6983, bisindolylmaleimide I, enzastaurin, sotrastaurin, and rottlerin have limited toxicity to all tested cells, consistent with previously reported observations (31, 32).

Our study demonstrated that viral replication is effectively blocked by all five PKC inhibitors in a SARS-CoV-2 replicon system and a wild-type virus infection model. Interestingly, the replication of the SARS-CoV-2 replicon is more susceptible to drug treatment than wild-type virus, validating the high efficiency of the replicon system.

In the kinetics test, the luciferase activities of the replicon system were significantly decreased by PKC inhibitors at 6 h posttransfection (Fig. 4A), suggesting that these drugs function at an early step of viral replication, such as protein translation or cleavage of the polyprotein. The protein translation of SARS-CoV-2 starts with a cap-dependent translation mechanism, in which multiple eukaryotic initiation factors such as the eIF4F complex are involved (33). In the eIF4F complex, a central scaffolding protein, eIF4G, is a substrate of PKCα (34). Therefore, we propose that the PKC inhibitors possibly impair the protein translation initiation of SARS-CoV-2 by reducing eIF4G phosphorylation. In contrast, the protein synthesis of ZIKV can be initiated in both cap-dependent and cap-independent ways, so the replication of the ZIKV replicon was not blocked by PKC inhibitors.

Another possible mechanism is that PKC inhibitors interfere with the cleavage of the viral polyprotein, similar to ebselen, which reduces the luciferase activity of the SARS-CoV-2 replicon at 6 h posttransfection (Fig. 4E). This observation is supported by a recent report showing that another PKC inhibitor, bisindolylmaleimide IX, targets the viral protease 3CL^pro^ in an *in vitro* test (21). Although PKC inhibitors do not directly inhibit the protease activity of 3CL^pro^ (Fig. 4F), they might indirectly impair its activity by inhibiting some PKC members involved in the 3CL^pro^ action. It should be pointed out that the possibility that PKC inhibitors disturb a late step of viral replication could not be completely ruled out, as the reduction in luciferase activities was more prominent at 24 h than at 6 h.

Our study showed that PKC inhibitors block several steps of SARS-CoV-2 infection, including the entry step (see Fig. S2 in the supplemental material) and protein translation (Fig. 4A and Fig. 5D). This finding is not unexpected as PKCs have diverse effects on infections by other viruses, through phosphorylating either viral proteins (such as DENV NS5) (19) or cellular proteins. PKC members play an antiviral role during infection by viruses (such as Rift Valley fever virus [RVFV], herpes simplex virus 1 [HSV-1], human papillomavirus 8 [HPV-8], and CHIKV) or promote infections by many viruses (such as HIV-1, MVM, and WNV). As Go 6983, bisindolylmaleimide I, enzastaurin, and sotrastaurin are pan-PKC inhibitors, we still need to determine which PKC isozyme(s) is the key member that supports SARS-CoV-2 infection by phosphorylating the target protein(s).

In conclusion, our study uncovered that several PKC inhibitors, including Go 6983, bisindolylmaleimide I, enzastaurin, sotrastaurin, and rottlerin, confer a potent ability to inhibit the replication of SARS-CoV-2, with limited cytotoxicity. To accelerate the clinical application of the PKC inhibitors, the roles of other PKC inhibitors and the impacts of PKC inhibitors on the replication, transmission, and pathogenesis of SARS-CoV-2 in *in vivo* animal models need to be investigated in the future.

## MATERIALS AND METHODS

### Cells and virus.

Baby hamster kidney cells (BHK-21), human hepatoma cells (Huh7), human lung carcinoma epithelial cells (A549), human embryonic kidney cells (HEK293T), and African green monkey kidney cells (Vero E6) were maintained in Dulbecco modified Eagle medium (DMEM) (Gibco) supplemented with 10% fetal bovine serum (FBS) (Gibco) at 37°C with 5% CO_2_. The media were supplemented with 100 U/mL of streptomycin and penicillin (Invitrogen, CA, USA). The ACE2-expressing A549 (A549-ACE2) cell lines were generated by transduction with a lentiviral vector (lentivector) expressing the human *ACE2* gene, as described previously (35). All cell lines were tested routinely and were free of mycoplasma contamination. The SARS-CoV-2 nCoV-SH01 strain (GenBank accession no. MT121215) stock (35) was prepared in Vero E6 cells. All experiments involving SARS-CoV-2 were performed in the biosafety level 3 (BSL-3) facility of Fudan University according to the rules of the BSL3 laboratory.

### Inhibitors.

Remdesivir (catalog no. SF-1193) was purchased from Beyotime (Shanghai, China). Go 6983 (catalog no. HY-13689), bisindolylmaleimide I (catalog no. HY-13867), enzastaurin (catalog no. HY-10342), sotrastaurin (catalog no. HY-10343), rottlerin (catalog no. HY-18980), and ebselen (catalog no. HY-13867) were purchased from MedChemExpress (MCE) (Shanghai, China).

### Antibodies.

Primary antibodies included anti-α-tubulin (BBI Life Science) and anti-GFP (Santa Cruz Inc.). Secondary antibodies for the immunofluorescence assay (IFA) included goat anti-rabbit secondary antibody (Alexa Fluor 488) and goat anti-mouse secondary antibody (Alexa Fluor 647) from Invitrogen.

### Replicon RNA transcription and transfection.

The pBAC-SARS-CoV-2-replicon-Luciferase plasmid was generated by Rong Zhang’s laboratory. The replicon sequence was derived from the SARS-CoV-2 SH01 strain. The replicon RNA was transcribed by using the mMESSAGE mMACHINE T7 transcription kit (catalog no. AM1344; Thermo Fisher Scientific) according to the manufacturer’s instructions. The DNA templates were removed by adding Turbo DNase and incubating the mixture at 37°C for 15 min. RNA was extracted by LiCl precipitation. The pelleted RNA was washed once with 70% ethanol, air dried, and gently resuspended in 100 μL RNase-free water. The replicon RNA was then stored at −80°C. BHK-21 or Huh7 cells were seeded onto 96-well plates 16 h before transfection. For each well, 20 ng replicon RNA and 0.12 μL Lipofectamine 2000 reagent (Invitrogen) were used according to the manufacturer’s instructions.

### Cell viability assay.

Cell viabilities were analyzed using cell counting kit 8 (CCK8) (catalog no. HY-K0301; MCE) according to the manufacturer’s instructions. Ten microliters of the CCK8 reagent was added to each well of a 96-well plate. After incubation at 37°C for 1 h, optical density (OD) values were measured at 450 nm using a BioTek instrument. The CC_50_ was calculated by nonlinear regression analysis.

### Luciferase assay.

The supernatants were cultured in a mixture of Nano-Glo luciferase buffer and the substrate (catalog no. N1120; Promega) (100:1) at room temperature for 15 min. All of the liquid was transferred to an opaque plate, and the chemiluminescence value was determined using a GloMax 96 luminometer (Promega).

### IC_50_ measurement.

Cells were pretreated with the indicated inhibitors at different concentrations 1 h prior to replicon RNA transfection or virus infection. The drugs were maintained in the media throughout the experiment. Luciferase reporter assays were performed at 24 h posttransfection or postinfection (p.i.).

### Western blotting.

Cells were lysed in radioimmunoprecipitation assay (RIPA) lysis buffer (pH 7.4) (50 mM Tris-HCl, 0.5% Nonidet P-40, 1% Triton X-100, 150 mM NaCl, 1 mM EDTA, 1 mM phenylmethanesulfonyl fluoride, 1% protease inhibitor mixture, 1 mM sodium orthovanadate, and 1 mM sodium fluoride). Proteins were separated on an SDS-PAGE gel and transferred onto nitrocellulose membranes, followed by blocking in 0.1% phosphate-buffered saline (PBS)–Tween (PBST) with 5% bovine serum albumin (BSA) and incubation with primary antibodies at 4°C overnight. Detection was performed with IRDye 800 CW-conjugated anti-rabbit IgG and IRDye 680 CW-conjugated anti-mouse IgG secondary antibodies (Li-Cor) or horseradish peroxidase-conjugated secondary antibodies (Bio-Rad). Immunoreactive bands were visualized using an Odyssey infrared (IR) imaging system (Li-Cor). The bands were quantified using Quantity One software (Bio-Rad).

### SARS-CoV-2 3CL^pro^ activity measurement.

SARS-CoV-2 3CL^pro^ activity was measured with a 2019 nCoV M^pro^/3CL^pro^ inhibitor screening kit (catalog no. P0312S; Beyotime) according to the manufacturer’s instructions. One microliter of SARS-CoV-2 3CL^pro^ reagent, 2 μL of the substrate, 5 μL of an inhibitor solution, and 93 μL of assay buffer were added to each well of a 96-well plate. After incubation at 37°C for 5 min, luciferase activities were measured at 490 nm using a multimode plate reader (Victor Nivo 5S).

### Pseudotyped virus assay.

Pseudoviruses were packaged in HEK293T cells by cotransfecting the retroviral vector (retrovector) pMIG (kindly provided by Jianhua Li, Fudan University) in which the target gene was replaced with the nanoluciferase gene, a plasmid expressing murine leukemia virus (MLV) Gag-Pol, and pcDNA3.1 expressing SARS-CoV-2 spike genes or vesicular stomatitis virus G (VSV-G) (pMD2.G; Addgene plasmid 12259) using Fugene HD transfection reagent (Promega). At 48 h posttransfection, the supernatant was harvested, clarified by spinning at 3,500 rpm for 15 min, aliquoted, and stored at −80°C. Virus entry was assessed by the transduction of pseudoviruses in 96-well plates. After 16 h, the luciferase activity was determined using a Nano-Glo luciferase assay kit (catalog no. N1110; Promega) according to the manufacturer’s instructions. The same volume of reagent was added to each well, and the mixture was shaken for 2 min. After incubation at room temperature for 10 min, luminescence was recorded by using a FlexStation 3 instrument (Molecular Devices), with an integration time of 1 s per well.

### Immunofluorescence assay.

Virus-infected cells were washed twice with PBS, fixed with 4% paraformaldehyde (PFA) in PBS for 30 min, and permeabilized with 0.2% Triton X-100 for 1 h. Cells were then incubated with in-house-made mouse anti-SARS-CoV-2 nucleocapsid protein serum (1:1,000) at 4°C overnight. After three washes, cells were incubated with the secondary goat anti-mouse antibody conjugated with Alexa Fluor 555 (catalog no. A-21424; Thermo) (2 μg/mL) for 2 h at room temperature, followed by staining with 4′,6-diamidino-2-phenylindole (DAPI). Images were collected using an Operetta high-content imaging system (PerkinElmer) and processed using PerkinElmer Harmony high-content analysis software v4.9 and ImageJ v2.0.0.

### Determination of SARS-CoV-2 RNA levels.

Cells were pretreated with 10 μM the inhibitor 1 h before SARS-CoV-2 infection (MOI = ~0.8). The drugs were maintained in the media throughout the experiment. At 24 h p.i., supernatant RNA was extracted using TRIzol reagent (Invitrogen) and reverse transcribed using Hi Script Q RT SuperMix (Vazyme) according to the manufacturer’s protocol. qRT-PCR was performed using LightCycler 480 SYBR green I master mix (Roche) in a CFX96 real-time system (Bio-Rad). Data were analyzed by using Δ*C_T_* values as described previously (36). The viral load was expressed on a log_10_ scale as viral RNA copies per milliliter of the supernatant. The primers and probes used are as follows: nCoV-N-Fwd (5′-GACCCCAAAATCAGCGAAAT-3′), nCoV-NRev (5′-TCTGGTTACTGCCAGTTGAATCTG-3′), nCoV-N-Probe (5′-FAM [6-carboxyfluorescein]-ACCCCGCATTACGTTTGGTGGACC-BHQ1 [black hole quencher 1]-3′), hGAPDH-Fwd (5′-TGCCTTCTTGCCTCTTGTCT-3′), hGAPDH-Rev (5′-GGCTCACCATGTAGCACTCA-3′), and GAPDH-Probe (5′-FAM-TTTGGTCGTATTGGGCGCCTGG-BHQ1-3′).

### Statistical analysis.

All experiments were independently repeated at least three times. Comparisons between two groups were performed using analysis of variance (ANOVA) with Dunnett’s multiple-comparison test or unpaired, two-tailed Student’s *t* tests. Graphs were generated using GraphPad Prism 8.0 software. *P* values of 0.05 or lower were considered to be statistically significant.
