# Supplementary material for: Protein Kinase C Inhibitors Reduce SARS-CoV-2 Replication in Cultured Cells
Source: Microbiol Spectr. 2022 Aug 24;10(5):e01056-22. doi: 10.1128/spectrum.01056-22 (PMC9603170; doi:10.1128/spectrum.01056-22)

Fig. S1. Chemical structures of PKC inhibitors.

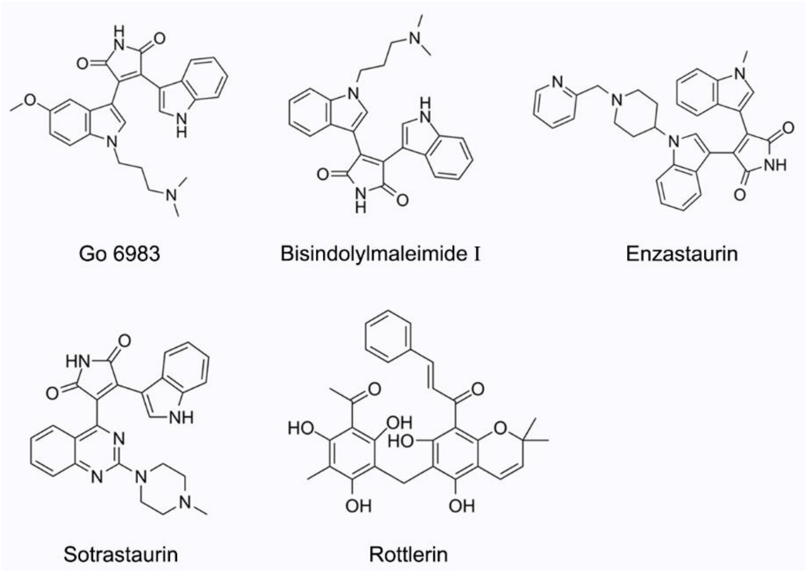

Fig. S2. Effect of PKC inhibitors on the virus entry. Cells were treated with DMSO, Go 6983, bisindolylmaleimide I, enzastaurin or sotrastaurin for 1 h, followed by pseudovirus infection. The luciferase activity of pseudovirus was measured at 16 h p.i.. Data were presented as mean ± standard deviation (SD) from three experiments. P values were calculated by ANOVA with Dunnett's multiple comparison test; ****p < 0.0001.

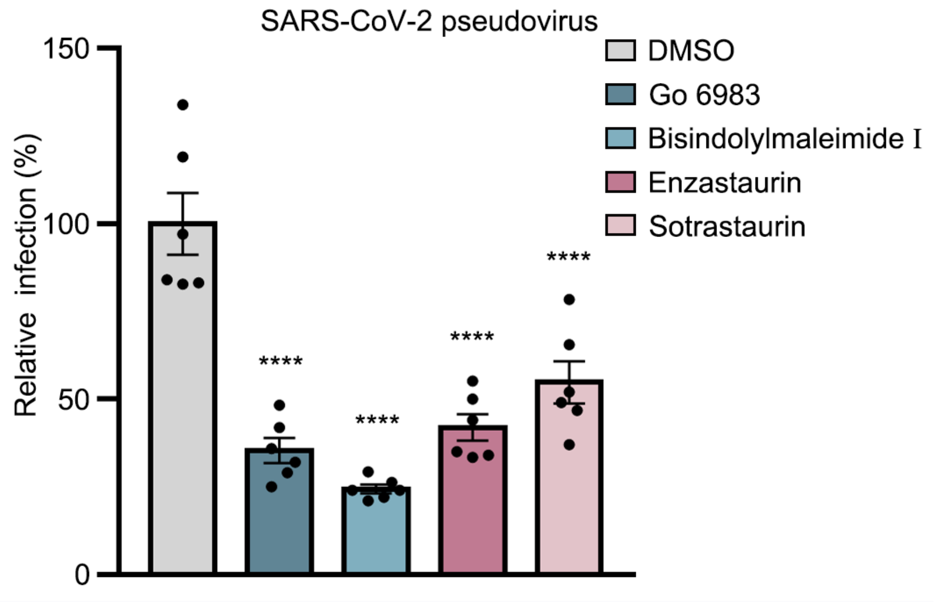

Supplement: Supplemental file 1 — Fig. S1 and S2. Download spectrum.01056-22-s0001.pdf, PDF file, 0.2 MB [file spectrum.01056-22-s0001.pdf]
